# A loop-mediated isothermal amplification (LAMP) assay for the detection of *Cryptotermes brevis* West Indian drywood termite (Blattodea: Kalotermitidae)

**DOI:** 10.1038/s41598-022-18582-1

**Published:** 2022-09-06

**Authors:** Vera Andjic, Aaron Maxwell, Michael Gorton, Diane M. White

**Affiliations:** 1 Department of Agriculture, Fisheries and Forestry, Perth, WA 6105 Australia; 2 Department of Agriculture, Fisheries and Forestry, Cairns, QLD 4870 Australia; 3 College of Science, Health, Engineering and Education, Murdoch University, Perth, WA 6150 Australia

**Keywords:** Biological techniques, Molecular biology

## Abstract

*Cryptotermes brevis* is one of the most destructive invasive termites in the subtropics and tropics and is a common biosecurity intercept at the Australian border. Drywood termite species are cryptic and difficult to identify morphologically in situations when soldiers or imagos are unavailable. We developed a novel DNA based loop-mediated isothermal amplification (LAMP) assay to detect *C. brevis* and differentiate it from other drywood termites. Validated voucher specimens of 30 different drywood termite species were obtained from several insect collections from which DNA was extracted and amplified. The amplicons containing partial mitochondrial 16S rRNA were sequenced and a DNA database was created from which *C. brevis* LAMP primers were developed, optimized, and tested. The assay was assessed against a range of target and non-target species and found to be specific, successfully amplifying the target specimens of *C. brevis* in under 30 min. Amplification success was variable against *C. brevis* faecal pellets due to minute, unmeasurable or degraded DNA. This LAMP test is a new tool for the rapid detection of *C. brevis* that will enable faster and less destructive management of drywood termite infestations.

## Introduction

*Cryptotermes brevis* (Walker) is an invasive species of termite in the order *Blattodea,* family *Kalotermitidae*, commonly known as the West Indian drywood termite. It is the most economically significant and widespread drywood termite in the tropics and subtropics due to its ability to infest soft and hardwoods in the absence of free water, damaging buildings and timber structures^1^. They can form colonies, feed, and reproduce in single pieces of wood enabling survival and transport in furniture, picture frames, and vessels^2–4^. *Cryptotermes brevis* is a regulated pest under the Australian *Biosecurity Act* 2015, is listed 25th of the 42 National Priority Plant Pests (NPPP) in Australia National Priority Plant Pests (2019)^5^ and, is a target for border inspections and surveillance^6^.

*Cryptotermes brevis* is one of the most intercepted drywood termites in imported timber products at the Australian border, and despite biosecurity control measures has been detected post border infesting timber structures including buildings. Quarantine measures to eradicate this destructive pest have been applied in restricted areas in Queensland and New South Wales where *C. brevis* was introduced^7–9^. Although this species was eradicated from New South Wales it remains in restricted areas in Queensland where it is a regulated pest under the Queensland Biosecurity Act 2014^9^. Costs to control infestations are significant, with the Queensland Government fumigating 600 buildings since 1976 at an average annual cost of $500,000^10^. The economic impact in the USA is estimated at over $100 million each year to control, and in the Azores its impact is estimated at €175 million^3,11^.

The identification of *C. brevis* heavily relies on morphology and morphometric analysis of soldiers and alates which only make up a small proportion of the termite colony and in their absence, species identification is not possible^12,13^. Colonies of *C. brevis* are difficult to detect in imported timber products and the only sign of their existence may be faecal pellets or “kick-out” holes. Procuring specimens essential for identification frequently involves destruction of timbers. This may be cost prohibitive for importers and may only recover pseudergates and damaged specimens for which morphometric identification is impossible. Consequently, imported goods are sent for fumigation to prevent pest spread and establishment. There is a need for a fast accurate molecular technique to distinguish *C. brevis* from other non-quarantinable and quarantinable drywood termites.

To date, the only existing molecular tool for the identification of *Cryptotermes* species are polymerase chain reaction (PCR) and DNA sequencing methods. These rely on specimens from which good quality DNA can be extracted, and the availability of sequences on public databases such as GenBank and Barcode of Life Data (BOLD). Time to identification can be several days depending on commercial sequencing services. Loop-mediated isothermal amplification (LAMP) is an alternative molecular tool that can be species-specific and deliver a result withing 30–60 min. An additional potential benefit to LAMP-based techniques is the identification of termites from faecal pellets. Pellets may contain minute amounts of termite DNA, although extracting the DNA and subjecting it to PCR is problematic. This is due to a lack of sensitivity of current PCR methods to the amount of target DNA present in pellets, high levels of PCR inhibitors, and DNA contamination from micro-organisms present in pellets. LAMP is a DNA-based method using loop-mediated isothermal amplification, which is robust, portable, simple, rapid, taxon specific and less sensitive to contaminants. It is therefore suitable for pest detection in the field and does not require specialist molecular technicians to operate. The advantage of the LAMP method over PCR and DNA sequencing is its high specificity due to the use of six primers pairs, less sensitivity from sample inhibitors, and simplicity. LAMP tests have been used for the detection of invasive insect pests^14–16^ and diseases^17–19^. The primary objective of this study was to develop a specific LAMP assay for the detection of *C. brevis* and their pellets.

## Results

### DNA extraction

The average yield of total DNA per individual termite was 3.96 ± 1.09 (SD) ng/µL, range 1.03–7.71 ng/µL. The DNA concentration from pellets was very low ranging from 0.07 to 0.3 ng/µL or was not measurable (Table 1).

**Table 1:** Designation of species, insect collection, and specificity test result of specimens used in LAMP test: *ANIC* Australian National Insect Collection, Commonwealth Scientific and Industrial Research Organisation, Canberra, Australia, *UFTC* University of Florida Termite Collection, The University of Florida*,* Institute of Food and Agricultural Sciences, USA, *FFPRI* Forestry and Forest Products Research Institute, Japan.

Taxon	Samples	Specimen code	Collection site and source	GenBank accession no	DNA concentration ng/µl	Peak Value (C°)	LAMP results	Time (min)
*Cryptotermes brevis*	Termite head	HI 49.0	USA (UFTC)	MT535915	3.9	80.5	+ ve	15:30
	Termite head	SA 134.0	South Africa (UFTC)	MT535974	4.08	80.3	+ ve	12:45
	Termite head	HN 688.0	Honduras (UFTC)	MT535978	2.79	80.6	+ ve	15:45
	Termite head	GUA 551.0	Guatemala (UFTC)	MT535979	7.71	80.7	+ ve	14:45
	Termite head	10–001,243	Unknown (ANIC)	MT535992	1.03	80.6	+ ve	23:30
	Pellets				unmeasurable	81.0	− ve	48.05
	Termite head	USA 2	USA (UFTC)	MT535997	4.19	79.9	+ ve	16:00
	Pellets				0.07	80.6	− ve	30.15
**Positive control**	**Termite head**	**USA 1**	USA (UFTC)	MT535995	19.1	80.8	+ ve	9:15
	Pellets			NA	0.3	80.5	+ ve	26:30
**Biological positive control**	Termite head	USA 1–1	USA (UFTC)	MT535995	19.1	80.7	+ ve	15:00
		USA 1–2			1.9	80.8	+ ve	16:15
		USA 1–3			0.19	80.7	+ ve	20:15
		USA 1–4			0.019	80.7	+ ve	24:45
		USA 1–5			0.0019	80.8	+ ve	25:45
		USA 1–6			0.00019		ND	
**Positive control**	gBLOCK	gBlock G1	ITD DNA Gene fragment	NA	10.0	80:6	+ ve	07:30
		gBlock G2			1.0	80:6	+ ve	09:00
		**gBlock G3**			0.1	**80:7**	+ ve	**10:15**
		gBlock G4			0.001	80.7	+ ve	11:45
		gBlock G5			0.0001	80.7	+ ve	16:15
		gBlock G6			0.00001	80.5	+ ve	20:00
*Cryptotermes domesticus*	Termite head	GA 26.0	USA (UFTC)	MT535999	1.62	NA	− ve	NA
	Termite head	ASA 202.0	USA (UFTC)	MT536003	1.03	NA	− ve	NA
	Pellets	unknown	Unknown (ANIC)		unmeasurable	NA	− ve	NA
*Cryptotermes dudleyi*	Termite head	AFR 1991.0	Nigeria (UFTC)	MT535964	3.3	NA	− ve	NA
	Termite head	TT 1073.0	Trinidad/Tobago (UFTC)	MT535966	2.56	NA	− ve	NA
	Pellets	NA	Malaysia	NA	unmeasurable	NA	− ve	NA
*Incisitermes minor*	Termite head		Japan (FFPRI)	NA	3.41	NA	NA	NA
	Pellets				unmeasurable	NA	− ve	NA

+ *ve* positive, − *ve* negative, *NA* Not available, *ND* Not detected. Positive controls are in bold.

### Development and assessment of the *C. brevis* LAMP assay

#### DNA sequence analysis and LAMP primer design

**Figure 1:**
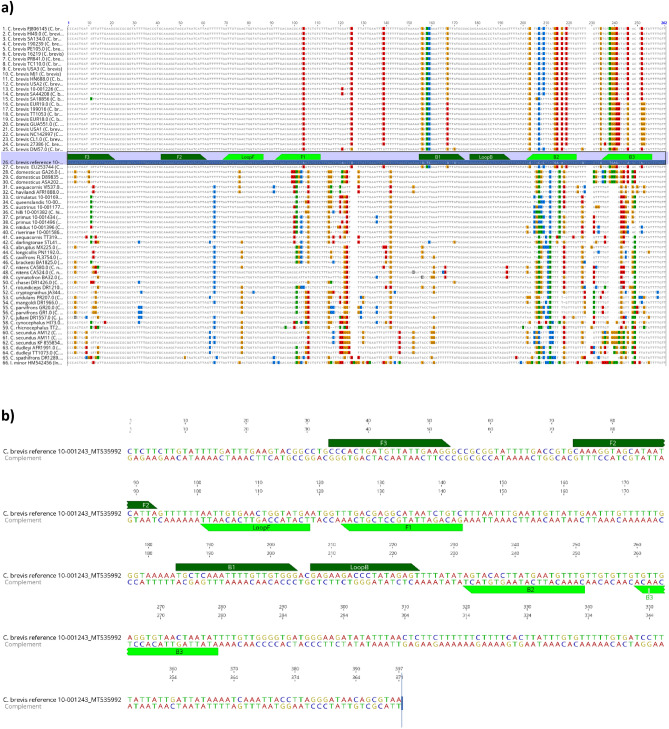
Alignment and location of primers and primer binding regions on partial sequence of mt16s rRNA sequences (**a**) Alignment of mt16s rRNA sequences of *C. brevis* and other *Cryptotermes* species used for primer design. (**b**) The reference sequence *C. brevis* MT535992. Inner primer, FIP, consists of F1 (complementary sequences) and F2. Another inner primer, BIP, is also composed of B1 and B2 (complementary sequences); F3, forward outer primer; B3, reverse outer primer; LB, loop backward; LF, loop forward. Primer information is documented in Table 2.

**Figure 2:**
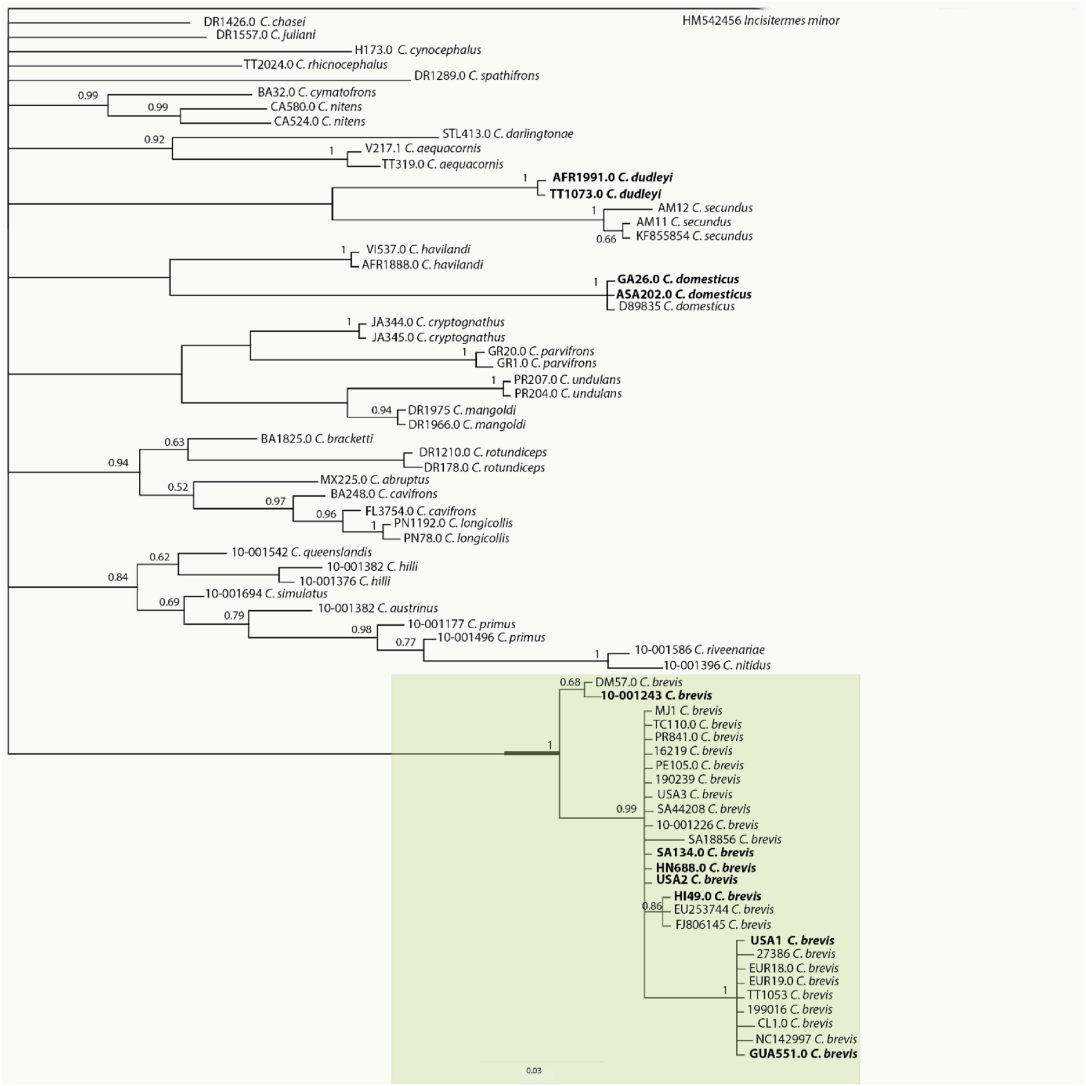
Bayesian phylogram of mt16S rRNA sequences showing the phylogenetic relationships of *Cryptotermes* species used in this study. Numbers represent Posterior probabilities for the nodes. Species tested in LAMP assay are in bold.

Novel LAMP primers (Table 2, Fig. 1a, b) were developed for the detection of *C. brevis* based on the DNA sequences of the partial mitochondrial 16S rRNA gene. The gene was successfully amplified across all *Cryptotermes* species used in the study (Supplementary Table S1). Based on phylogenetic analysis, none of the *Cryptotermes* species were very close to *C. brevis* (Fig. 2). The DNA sequence similarity between *C. brevis* and other *Cryptotermes* species ranged from 79 to 88%. The DNA sequences of the three most common intercepted species associated with wood in service that are morphologically similar to *C. brevis* were different: *C. domesticus* (81% identity), *C. dudleyi* (82%), *Incisitermes minor* (75.6%) (Supplementary Table S2). This gives confidence in the identification of these species and in the species-specific LAMP primers developed. The *C. brevis* LAMP assay consists of six primers, the outer forward primer F3, the outer reverse primer B3, the inner forward primer FIP, the inner reverse primer BIP, the reverse loop primer LoopF and the forward loop primer LoopB (Table 2). The optimal primers ratio was (F3/B3: FIP/BIP: LoopF/LoopB) 10: 1: 2. In addition, a 203 bp gBlock dsDNA fragment was created for use as synthetic DNA positive control for *C. brevis* LAMP assay (Table 2).

**Table 2:** LAMP primers sequence and concentration developed in this study.

Primer name	Primer sequences 5’ to 3’	Primer concentration in LAMP reaction (1X)	Primer length (bp)
***C. brevis*** **gBlock**	ATCATTAGTTTTTTAATTGTGAACTGGTATGAATGGTTTG ACGAGGCATAATCTGTCTTTAATTTGGATTATTATTGAAT TTATTTTTTGGGTAAAAATGCTCAAATTTTGTTATGGGAC GAGAAGACCCTATAGAGTTTTATATAGTACACTTATGAGT ATTTGTTTTGTTGTGTTGGAGTGTAACTAATATTTTGTTG GGG		203
***C. brevis*** **F3**	CCCACTGATGTTATTGAAGG	0.08 µM	20
***C. brevis*** **B3**	ATATTAGTTACACCTCAACA	0.08 µM	20
***C. brevis*** **FIP (F1c + F2)**	GACAGATTATGCCTCGTCAACAAAGGTAGCATAATCATTA	0.8 µM	40
***C. brevis*** **BIP (B1c + B2)**	TGCTCAAATTTTGTTGTGGGCAAACATTCATAAGTGTACT	0.8 µM	40
***C. brevis*** **LoopF**	TCATACCAGTTCACAATT	0.4 µM	18
***C. brevis*** **LoopB**	GAGAAGACCCTATAGAGT	0.4 µM	18

*F3* forward outer primer, *B3* reverse outer primer, *FIP* forward inner primer (comprising F1c and F2 sequences), *BIP* reverse inner primer (comprising B1c and B2 sequences), *LB* loop backward, *LF* loop forward. Sequences F2 and B2 are underlined.

#### In-silico analysis

The phylogenetic analysis did not reveal any closely related *Cryptotermes* species to the target species *C. brevis. In-silico* analysis of the primer binding sites indicated specificity exclusively for *C. brevis* with none of the other 30 species tested indicating potential for binding. Therefore, the LAMP assay was tested in-vitro on the target species and three other morphologically similar and commonly intercepted species at the Australian border. The test included six representative termite specimens of *C. brevis*, two *C. domesticus*, two *C. dudleyi,* and one *I. minor*. The assay was also tested on the pellet samples of these species (Table 1).

### Specificity and sensitivity of LAMP assay

The specificity of the LAMP assay was tested on six representatives of *C. brevis* (alates, soldiers and pseudergates) and two morphologically similar species (as pseudergates or imagos): two representatives of *C. domesticus*, two of *C. dudleyi;* and one representative of the herterogeneric *I. minor*. The primers were also tested on the pellets of all three species (four *C. brevis* including positive control, and one of each pellet of; *C. domesticus*, *C. dudleyi*, *I. minor*).

**Figure 3:**
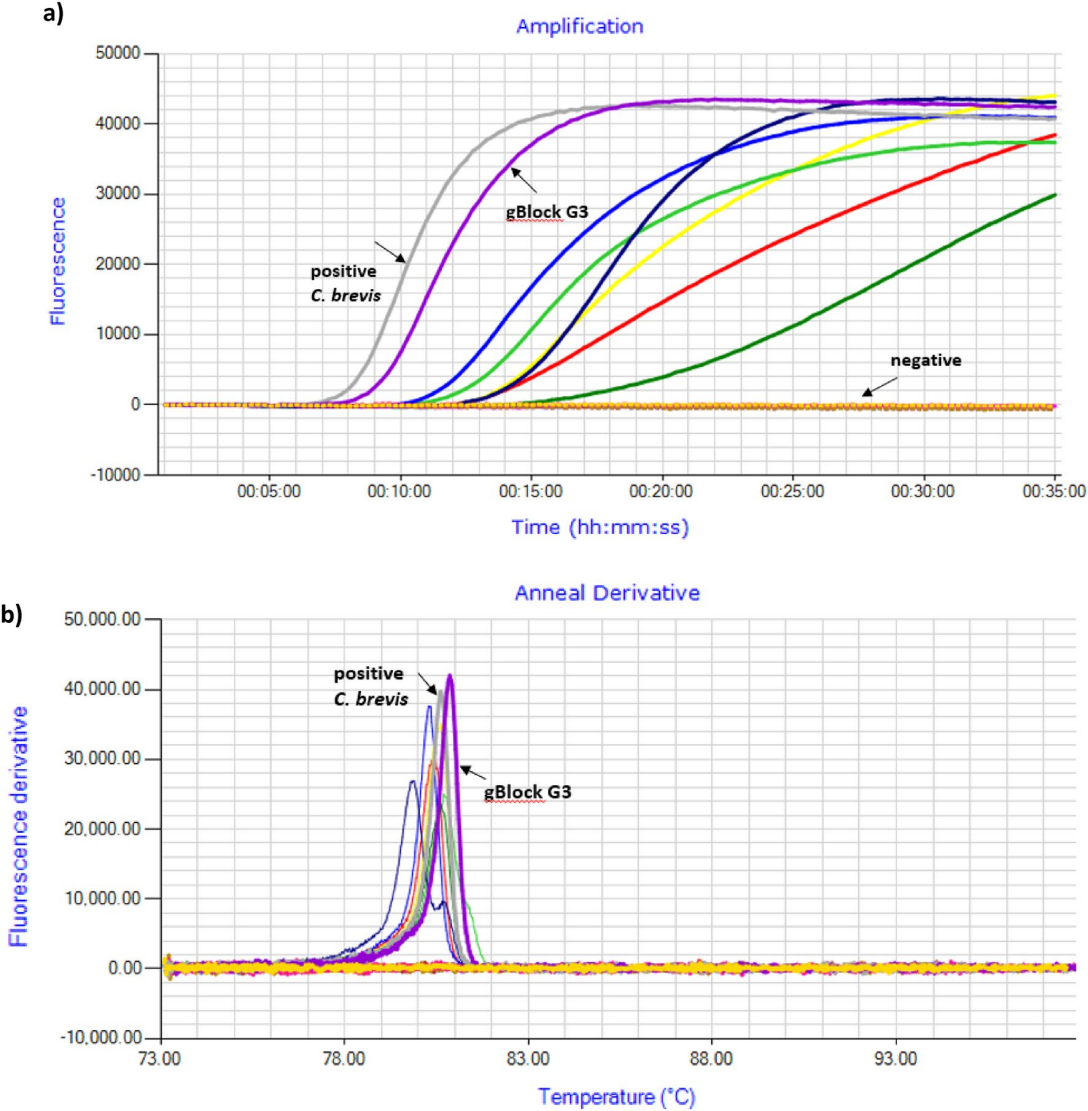
LAMP assay performed on DNA extracts of *C. brevis*, *C. domesticus*, *C. dudleyi* and *I. minor* specimens. (**a**) Amplification profile with eight positive samples amplifying in ≤ 30 min*;* positive = positive *C. brevis* control; G3 = synthetic positive control gBlock; negative = negative samples showing flat line. (**b**) Anneal derivative of LAMP amplicons with anneal derivative 80.5–80.8 °C.

The *C. brevis* LAMP assay detected all six tested *C. brevis* insect samples. The assay produced amplification of the target species in less than ≤ 30 min (Mean value 15.0 min, Standard Deviation ± 4.0) at anneal derivate 79.9–80.9 °C (Mean value 80.6 °C, Standard Deviation ± 0.4) (Table 1, Fig. 3a, b, Supplementary Table S3). Amplification peaks were tall and regular for four samples indicating efficient amplification. Two positive samples (HI 49.0-red peak and 10–001,243-dark green peak) had slightly irregular amplification peaks suggesting the possibility of DNA degradation (Fig. 3a). The *C. brevis* LAMP assay did not detect any cross-reactivity on the tested non-target species, only the positive controls (biological *C. brevis* and gBlock) produced an amplification peak after 09:15–10:15 min resulting in a negative LAMP test result (Table 1, Fig. 3a, b).

**Figure 4:**
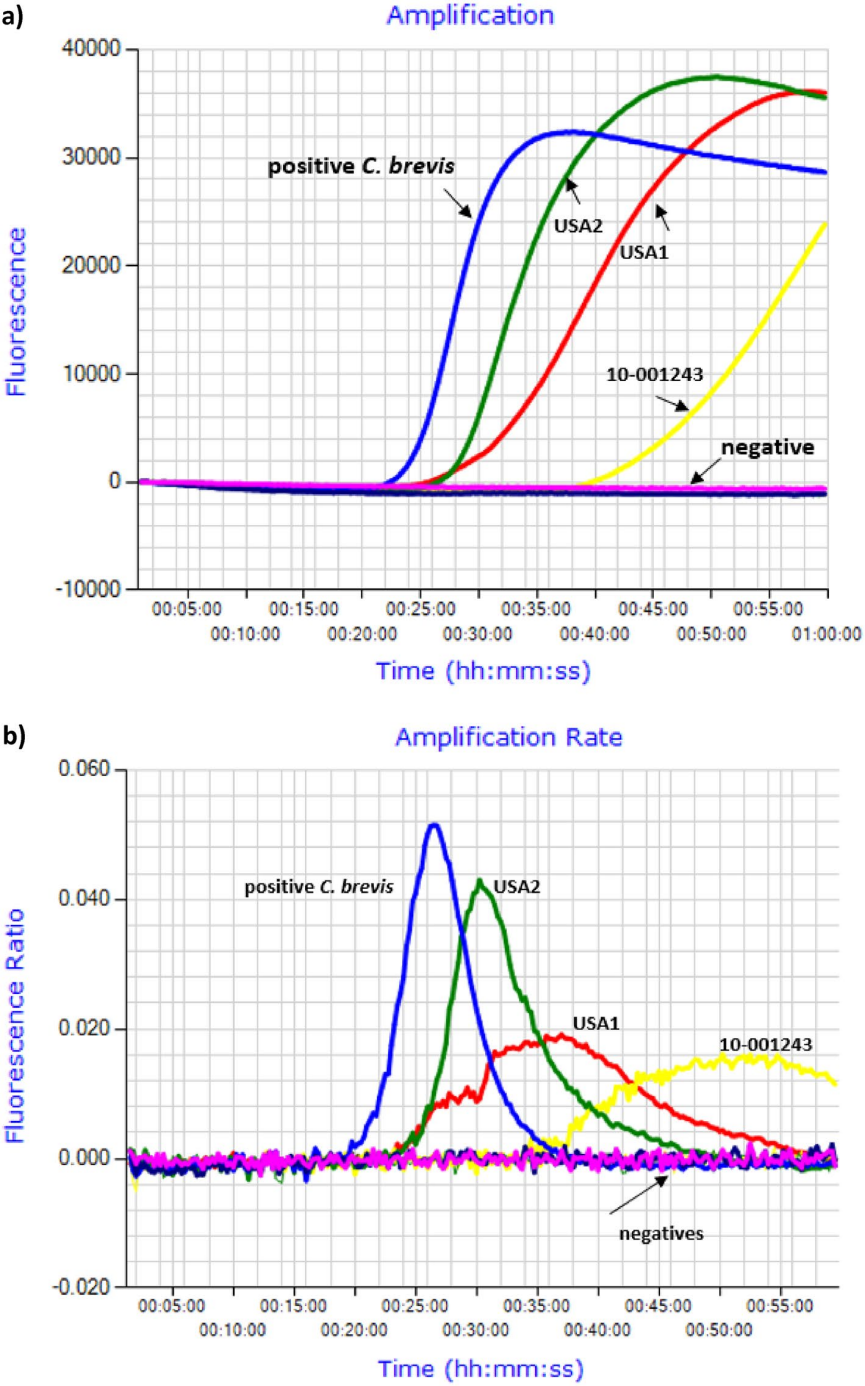
LAMP assay performed on DNA extracts from pellets samples of *C. brevis*, *C. domesticus*, *C. dudleyi,* and *I. minor*. (**a**) Amplification profile with positive samples amplifying from ≤ 30 min*;* positive = positive *C. brevis* control; negative = negative samples including negative control showing flat line. (**b**) Amplification rate of LAMP amplicons with anneal derivative 80.5–80.6 °C.

In testing pellet samples, the LAMP assay detected the positive control *C. brevis* pellet before time to positivity of 30 min (26.30 min). This was at annealing derivate 80.5 °C, with DNA concentration of 0.3 ng/µl. The three other *C. brevis* pellet samples (USA 1; USA 2, 10-001243) tested, showed late amplification. Because these samples amplified after 30 min (30.15, 36.09, 48.05) they were assigned as negative in this test. The DNA concentrations of those three samples were low (0.07 ng/µl) or unmeasurable. When compared to the positive control, the amplification rate peaks for the samples producing amplicons after 30 min were short and were irregular (Table 1, Fig. 4a, b). Pellets of the non-target species did not produce any amplification peaks (Table 1, Fig. 4a, b). The repeated LAMP assay on pellets has shown a similar pattern (Supplementary Tables S3, Fig. 2S1).

**Figure 5:**
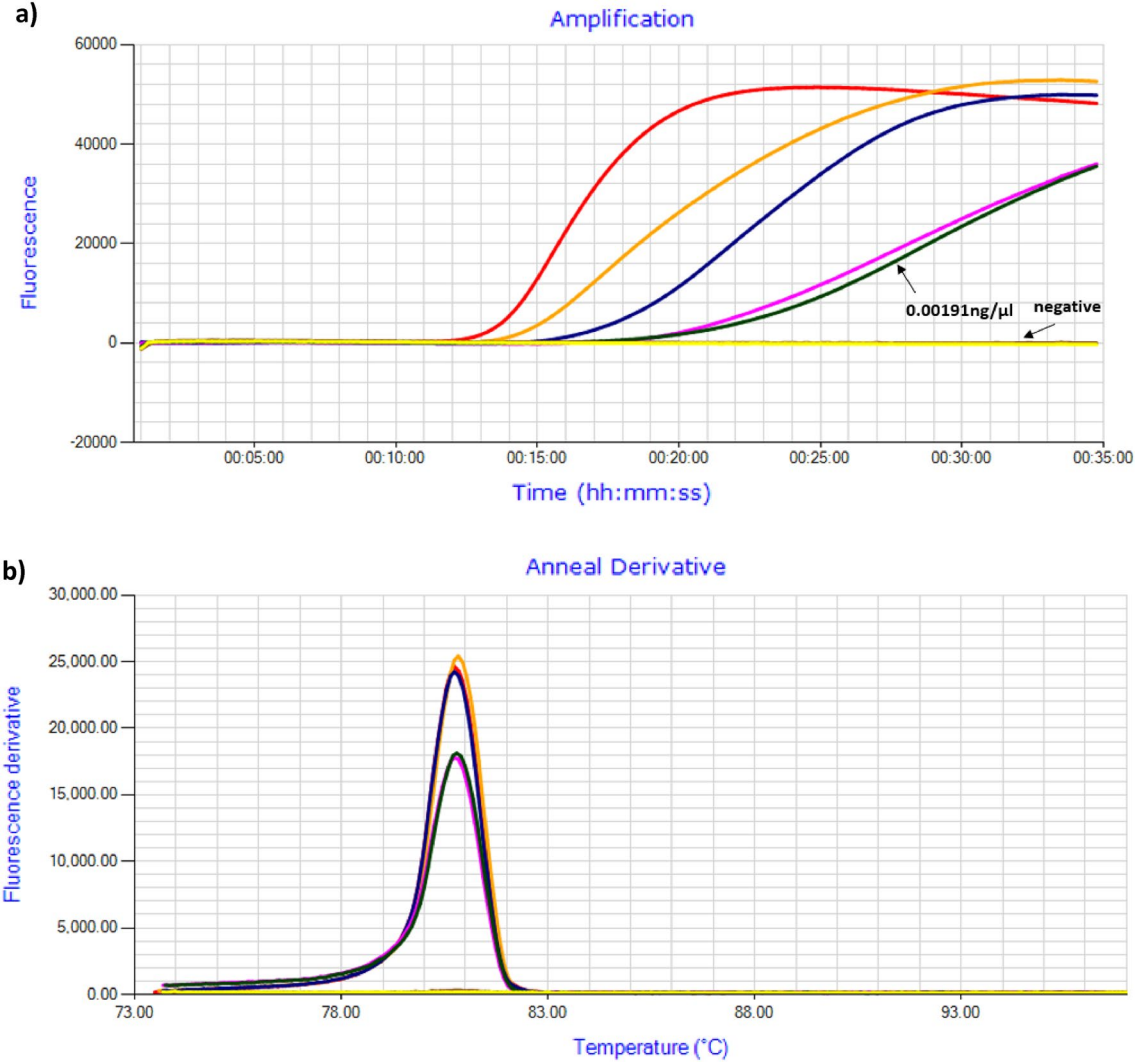
Detection sensitivity of *C. brevis* biological positive control amplicons. (**a**) Amplification profile with template concentration ranging from 19.1 to 0.00019 ng/µl at ten-fold dilution. (**b**) Annealing derivative of LAMP amplicons with an anneal derivatives of 80.7–80.8 °C.

The sensitivity of ten-fold serial dilutions of biological *C. brevis* positive control (USA1) showed positive amplification peaks after 15.00 to 25.45 min with annealing derivative temperature peak at 80.7 °C (Fig. 5a, b) and detection sensitivity of 0.0019 ng/µl (approximately 3 copies of DNA per µl) of DNA.

**Figure 6:**
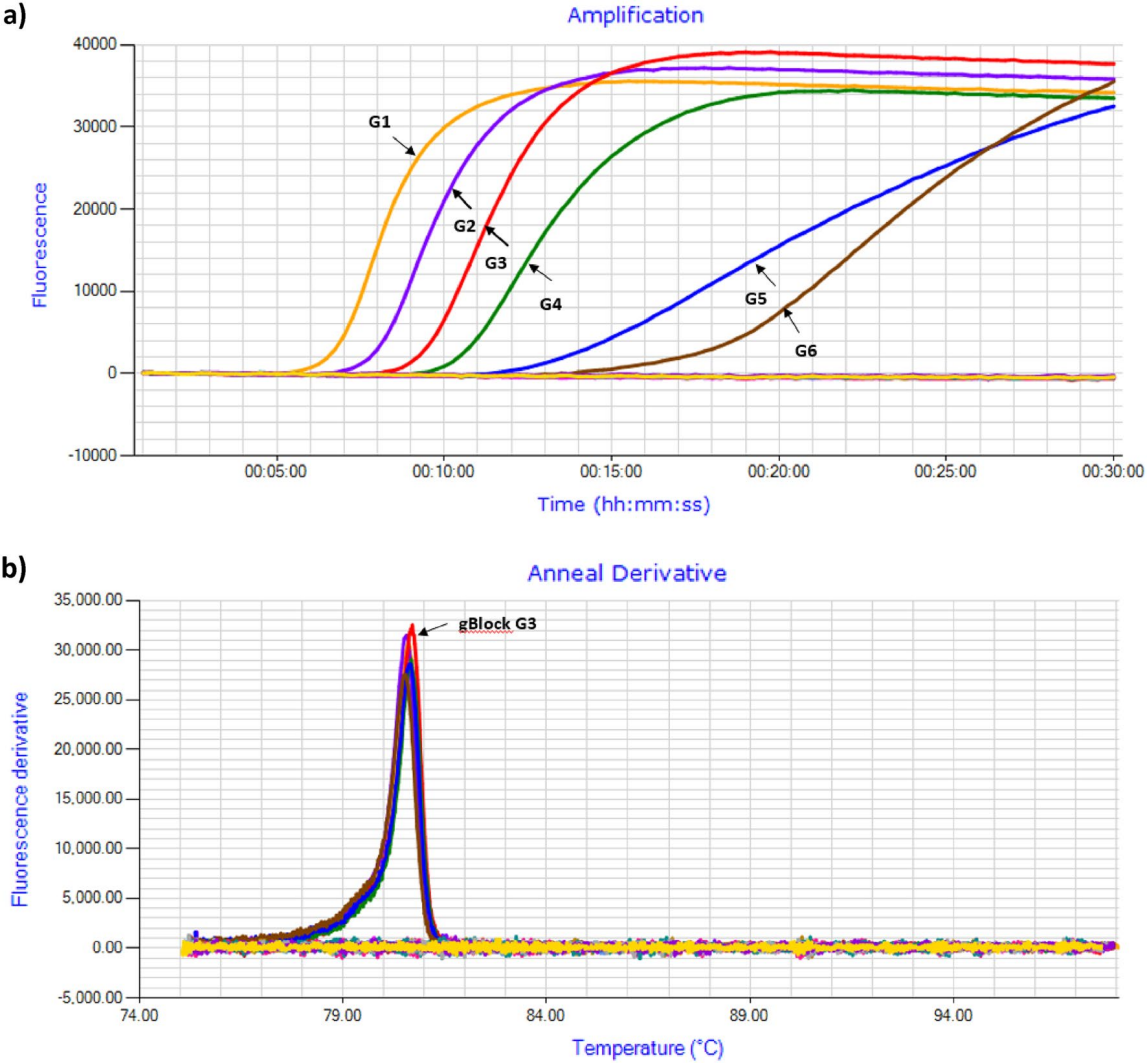
Performance of *C. brevis* gBlock dsDNA amplicons. (**a**) Amplification profile with DNA templates ranging from 10 to 0.00010 ng/µl at ten-fold dilution. (**b**) Annealing derivative of LAMP amplicons with an anneal derivatives of 80.5–80.7 °C.

The performance of gBlock was tested using ten-fold dilutions DNA concentration ranging from 10 to 0.00001 ng/µl in LAMP reactions with positive detections found after 7.30–20.00 min. The detection sensitivity of gBlock was 0.00001 ng/µl (G6) of DNA within 20 min (Fig. 6a). The gBlock anneal derivate peak occurred at 80.7 °C (Fig. 6b). Based on amplification time, reproducibility, and consistency we conclude that gBlock G1 to G4 could be alternatively used as positive control.

## Discussion

In this study, a novel *C. brevis* LAMP assay was developed and tested. The DNA sequences of the mt16S DNA gene were sufficiently variable to distinguish closely related *Cryptotermes* species including *C. domesticus*, *C. dudleyi,* and *I. minor* that are morphologically similar as pseudergates. Variability at this locus was sufficient to enable design of the *C. brevis* specific LAMP assay. The phylogeny of the mt16S gene revealed that the target termite, *C. brevis* is monophyletic, forming three sub-clades with slight sequence variation, all of which the primers were designed to detect. The LAMP primers were tested *in-silico* against all 30 species and in-vitro on close relatives (*C. domesticus*, *C. dudleyi,* and *I. minor*) that are difficult to distinguish as pseudergates and are most important for the purpose of Biosecurity decision making. The LAMP assay developed in this study is specific and able to detect the target species. No cross-reactions were observed in morphologically similar species, and this gives confidence in our *C. brevis* LAMP assay. Previously, the mitochondrial 16S gene has been successfully used in a LAMP assay for detection of pseudergates and faecal pellets of *Incisitermes minor*^14^. Although this gene is not the standard barcode for insects it was found to be the most informative for termites diagnostics in the present and previous studies^19,20^. We also designed a synthetic positive control "gBlock" that may be utilised in the *C. brevis* LAMP assay. This offers a reliable positive control that can be readily acquired from commercial gene fragment manufacturers without the challenges of obtaining biological DNA for positive control material. The synthetic positive control enables validation of each LAMP assay and gives confidence in the results obtained.

A few species of *Kalotermitidae*, including *C. brevis* are pre-adapted to invade new locations via anthropogenic global trade and movement, due to their evolved strategy to disperse via pieces of wood ^21,22^. First and foremost, mature colonies of pestiferous kalotermitids produce primary winged reproductives to found new colonies such as those from docked boats to lighted shore structures. Additionally, they produce secondary reproductive stages^23^, enabling residual colonies to establish in new locations^21^ and presenting significant biosecurity risk. Because of the difficulty in extracting suitable termite life-stages to identify *C. brevis* morphologically, molecular tools are crucial for managing the biosecurity risk. Currently, the only molecular biology diagnostic method for *C. brevis* is Sanger DNA sequencing method^24^ of the mt16S gene. This method requires significantly longer time and effort, up to 3–7 days, compared to 1–3 h for LAMP diagnostics. These time savings are critical in enabling a rapid response in managing Biosecurity risk. LAMP technology is becoming one of the most popular molecular methods for the detection of pests and diseases as it is a reliable, fast, and sensitive diagnostic tool. For instance, LAMP has been used in the early detection of pests and diseases such as *Phytophthora ramorum*^25^, *Xylella fastidiosa*^26^ *Bemisia tabaci*, *Thrips palmi*^27^, *Mythimna loreyi*^28^. Currently, the only LAMP assay developed for drywood termite detection is for *Incisitermes minor* developed by Ide et al.^14^.

In many cases termite pellets are the only sample that can be obtained without destructive sampling of goods of high value, such as timber containing vessels. In this study, the LAMP assay detected *C. brevis* from pellet samples. However, in some cases the amplification peaks were produced after 30 min which exceeds the acceptable detection time, therefore the results were called negative. Pellets of non-target species did not produce peaks. Rizzo et al.^16^ suggested that when developing an assay for pellet samples, three critical points must be considered: (1) insufficient amount of DNA in the sample; (2) the presence of inhibitors deriving from pellets and (3) the possibility of DNA degradation over the time. These factors are likely the cause of the late amplifications of *C. brevis* pellet samples. The DNA concentration was much lower in pellets (0.07–0.3 ng/µL) than in insect specimens (1.03–7.71 ng/µL) and some of the pellets were 20 years old. Also, pellets contain polysaccharides and other compounds known to inhibit PCR^29^. Ide et al.^14^ developed a visual LAMP test for *I. minor* and concluded that detection in pellets older than 3 months is inconsistent. Obtaining fresh pellets was very difficult because *C. brevis* is absent from most of Australia. Therefore, most of the pellet samples used in this study were from insect collections more than a year old. Kyei-Poku et al*.* 2020 used a LAMP technique for the detection of the emerald ash borer from pellets, and also concluded the amount of extracted DNA from insects was more stable and higher than in their pellet samples^30^.

This is the first LAMP detection method developed for *C. brevis*, one of the most damaging drywood termite pests in the world, and a high priority drywood termite species of biosecurity concern to Australia. The LAMP-based assay optimized in this study only takes three hours to complete the full diagnostic (DNA extraction, amplification, and annealing). The *C. brevis* LAMP assay developed in this study can be used for the detection of *C*. *brevis* termite specimens; but is not reliable for their detection from pellet samples. This LAMP assay will assist in managing *C. brevis* threats across the biosecurity continuum; and allow for faster detection and mitigation of risks in a range of situations where drywood termites have infested timber.

## Materials and methods

### Specimens examined

In total sixty-five *Cryptotermes* individuals containing 30 *Cryptotermes* species (including 25 specimens of *C. brevis*) and one *Incisitermes minor* were used in the initial study (Supplementary Table S1). Specimens were acquired from: Australian National Insect Collection (ANIC) CSIRO, Australia; University of Florida Termite Collection (UFTC); Forestry and Forest Products Research Institute Insect Collection (FFPRI), Japan; Department of Agriculture, Fisheries and Forestry, Australia, from border interceptions (DAFF). All specimens were morphologically verified by termite experts and by DNA sequencing and phylogenetic analysis of the partial mitochondrial mt16S rRNA gene region. The species were selected to include examples of the target species *C. brevis* and the closest relatives for testing the specificity of the LAMP assay, as well as the three morphologically similar and commonly intercepted species at the Australian border: *C. domesticus*, *C. dudleyi*, *I. minor* (Table 1).

### DNA extractions

Genomic DNA was extracted from ethanol preserved termite heads using the Qiagen QIAamp DNA Mini Kit (Qiagen, CA, USA) following the manufacturer’s recommendations. Genomic DNA from termite pellets was obtained using the BIOLINE ISOLATE II Plant DNA Kit (Bioline, London, UK) following the manufacturer’s protocol. The concentration of purified DNA solution was determined by Qubit 3.0 fluorometer (ThermoFisher Scientific Waltham, MA USA). The extracted DNA was used for molecular confirmation of the species used in this study by DNA sequence analysis of mt16S rRNA gene region and the LAMP assay.

### Development and assessment of the *C. brevis* LAMP assay

#### DNA sequence analysis and LAMP primer design

The mt16S rRNA sequences from forward and reverse (LR-J-13007^31^)/LR-N-13398^31,32^) primer pair reads were generated via De Novo Assembly implemented in Geneious Prime 2020 2.2 (https://www.geneious.com). The consensus sequences were subjected to BLASTn (megablast) searches (https://blast.ncbi.nlm.gov/Blast.cgi) to search for the closest relatives for *Cryptotermes* species and to confirm the identity of validated voucher specimens. Closely related sequences were downloaded in Geneious, aligned, and used for primer development. There were very few mitochondrial 16S ribosomal sequences of *Cryptotermes* species available from the GenBank database (only two *C. brevis* sequences FJ 806145, EU253744, none for *C. dudleyi*). Therefore, a local BLAST library was constructed using the sequences obtained in this study and the sequences from GenBank (Supplementary Table S1). This library was used for BLAST searches for this study. The consensus sequences were aligned using the MAFFT algorithm implemented in Geneious. Highly conserved regions within the sequence alignment targeting *C. brevis* were identified manually and assessed for their suitability for LAMP primer development (Fig. 1a). The LAMP primers were designed from a validated voucher specimen of *C. brevis* 10-001243 from which the mt16S rRNA sequences were obtained in this study (GenBank accession no. MT535992; Fig. 1b) using LAMP Designer software version 1.15 (Premier Biosoft International, USA) and confirmed by a local BLAST search to ensure their specificity (*in-silicoin-silico*). The phylogeny of *Cryptotermes* species was estimated using Bayesian analysis which was performed with MrBayes^33^ software implemented in Geneious Prime 2020 2.2 (https://www.geneious.com). Phylogenetic analysis was performed to estimate the relationship between closely related *Cryptotermes* species and *C. brevis* (Fig. 2).

#### LAMP assay conditions

Each LAMP reaction was performed in a total volume of 25 μl containing 15 μl of Isothermal Master Mix (ISO-001, OptiGene Ltd, UK), 10 µM stock of each primer F3 (0.2 μl), B3 (0.2 µl) FIP (2.0 μl), BIP (2.0 µl), LoopF (1.0 μl), LoopB (1.0 µl), 1.6 μl of PCR grade water and 2 μl of template DNA. A synthetic gene fragment dsDNA (gBlock) was generated from the fragment of *C. brevis* reference sequence MT535992 developed earlier by authors for detection of *C. brevis* by real time PCR and tested for reliability as a positive control (www.idtdna.com/pages/products/gene-and-gene-fragments). The DNA fragment consisted of 203 bases containing part of *C. brevis* mt16S gene including five LAMP primers regions (F3 not included) (Table 2). The DNA of a verified (by morphology and DNA Sequencing) biological *C. brevis* termite (Table 1: USA1) was used as a positive control in all LAMP tests. Each test included a biological positive control, a no template negative control (NTC), and test samples. The LAMP assays were run on the Genie II at 62 °C for 35 min) followed by an annealing curve analysis from 98 to 73 °C with ramping at 0.05 °C/s. To ensure reproducibility and accuracy, each LAMP assay was performed in triplicate and mean of peak values and annealing temperatures recorded (Supplementary Table S3).

### Specificity and sensitivity of LAMP assay

The LAMP assay was tested against the target species and checked for cross reactivity on morphologically related species (non-target species) *C. brevis* and *C. domesticus*, *C. dudleyi,* and *I. minor* (Table 1). After the run completion, the amplification, amplification rate, and anneal derivative curves were visualised and assessed using Genie Explorer software version 1.15 (www.optigene.co.uk/support). The time of amplification (minutes) and anneal derivative temperature (°C) were compared against positive controls to confirm that no false positive or negative was amplified (Table 1). The primer specificity was assessed following the manufacturer’s recommendations assessing the amplification time and amplification peaks. The samples showing traces in less or equal to 30 (≤ 30) minutes with regular peaks were called positive and one above 30 (> 30) with flat peaks were called negative. Non-target species and the negative control are expected to have flat amplification lines.

The detection sensitivity of *C. brevis* LAMP assay was assessed by preparing a ten-fold serial dilution of verified DNA of *C. brevis* termite (USA1). Starting DNA concentration of *C. brevis* was quantified using Qubit 3.0 fluorometer following the manufacturer protocol. The DNA sample was serially diluted at six concentration points ranging from 19.1 to 0.000191 ng/µl. The samples were run in the Genie II as described above. The initial quantification values from Qubit, were converted from nanogram per microlitre to copies per microlitre. Calculations were based on a *C brevis* genome size of 1.3 × 10^9^ base pairs as per *C. secundus* ^34^.

To test the performance of the synthetic positive control—gBlock, a ten-fold serial dilution of the gBlock DNA fragment was prepared using ultrapure water (Fisher Biotech, Australia). The DNA was diluted from 10 to 0.00010 ng/µl. The gBlock LAMP test was performed in the Genie II under the same LAMP assay conditions as previously described.

## Supplementary Information

Supplementary Information 1.

Supplementary Information 2.

Supplementary Information 3.

## Data Availability

GenBank, accession numbers in Supplementary Table 1.
