# Supplementary material for: A loop-mediated isothermal amplification (LAMP) assay for the detection of Cryptotermes brevis West Indian drywood termite (Blattodea: Kalotermitidae)
Source: Sci Rep. 2022 Sep 6;12:15111. doi: 10.1038/s41598-022-18582-1 (PMC9448757; doi:10.1038/s41598-022-18582-1)
Supplement: Supplementary file 1 — Supplementary Information 1. [file 41598_2022_18582_MOESM1_ESM.docx]

**Table S1.** List of specimens used in phylogenetic study. ANIC = Australian National Insect Collection, Commonwealth Scientific and Industrial Research Organisation, Canberra, Australia; UFTC=University of Florida Termite Collection, Institute of Food and Agricultural Sciences, USA; DAFF = Department of Agriculture, Fisheries and Forestry, Australia; FFPRI = Forestry and Forest Products Research Institute, Japan. Specimens used in LAMP testing are in bold.

| **Sample code** | **Taxon** | **Collection sites** | **GenBank Accession No.** |
| --- | --- | --- | --- |
| MX 225.0 | *Cryptotermes abruptus* | Mexico (UFTC) | MT535949 |
| TT 319.0 | *C. aequacornis* | Trinidad/Tobago (UFTC) | MT535951 |
| 10-001177 | *C. austrinus* | Australia (ANIC) | MT536011 |
| BA 1825.0 | *C. bracketti* | Bahamas (UFTC) | MT535935 |
| **HI 49.0**  **SA 134.0**  SA 44208  MJ1  27386  **HN 688.0**  **GUA 551.0**  SA 18856  DM 57.0  CL 1.0  NC 142997  TC 110.0  EUR 18.0  PR 841.0  **10-001243**  EUR 19.0  16219  **USA1**  PE 105.0  **USA2**  TT 1053.0  190239  USA3  199016  10-001226  ISO21  BYU IGS IS21 | *C. brevis* | USA (UFTC)  South Africa (UFTC)  South Africa (DAFF)  Unknown (DAFF)  Unknown (DAFF)  Honduras (UFTC)  Guatemala (UFTC)  South Africa (DAFF)  Dominica (UFTC)  Chile (UFTC)  New Caledonia (DAFF)  Turks and Caicos (UFTC)  Portugal (UFTC)  U.S. Territory (UFTC)  Unknown (ANIC)  Portugal (UFTC)  Unknown (DAFF)  USA (UFTC)  Peru (UFTC)  USA (UFTC)  Trinidad/Tobago (UFTC)  Unknown  Peru (UFTC)  Egypt (DAFF)  QLD, Australia (ANIC)  Unknown  Unknown | MT535915  MT535974  MT535976  MT535975  MT535977  MT535978  MT535979  MT535980  MT535986  MT535987  MT535988  MT535989  MT535990  MT535991  MT535992  MT535993  MT535994  MT535995  MT535996  MT535997  MT535998  MT536005  MT536006  MT536009  NA  FJ806145  EU253744 |
| FL 3754.0 | *C. cavifrons* | Monroe (UFTC) | MT535961 |
| DR 1426.0 | *C. chasei* | Dominican Republic (UFTC) | MT535960 |
| JA 344.0 | *C. cryptognathus* | Jamaica (UFTC) | MT535932 |
| BA 32.0 | *C. cymatofron* | Bahamas (UFTC) | MT535959 |
| HI 73.0 | *C. cynocephalus* | USA (UFTC) | MT535948 |
| STL 413.0 | *C. darlingtonae* | St. Lucia (UFTC) | MT535933 |
| **GA 26.0**  **ASA 202.0**  D89835 | *C. domesticus* | USA (UFTC  USA (UFTC)  Japan | MT535999  MT536003  D89835 |
| **AFR 1991.0**  **TT 1073.0** | *C. dudleyi* | Nigeria (UFTC)  Trinidad/Tobago (UFTC) | MT535964  MT535966 |
| AFR 1888.0 | *C. havilandi* | Gabon (UFTC) | MT535955 |
| 10-001382 | *C. hilli* | Australia (ANIC) | MT536017 |
| DR 1557.0 | *C. juliani* | Dominican Republic (UFTC) | MT535957 |
| PN 1192.0 | *C. longicollis* | Panama (UFTC) | MT535958 |
| DR 1966.0 | *C. mangoldi* | Dominican Republic (UFTC) | MT535954 |
| CA 524.0  CA 580.0 | *C. nitens* | Cayman Island (UFTC)  Jamaica (UFTC) | MT535956  MT535985 |
| 10-001396 | *C. nitidus* | Australia (ANIC) | MT536014 |
| GR 20.0  GR 1.0 | *C. parvifrons* | Grenada (UFTC) | MT535937  MT535942 |
| 10-001496  10-001434 | *C. primus* | Australia (ANIC)  Australia (ANIC) | MT536015  MT536019 |
| 10-001542 | *C. queenslandis* | Australia (ANIC) | MT536021 |
| TT 2024.0 | *C. rhicnocephalus* | Trinidad/Tobago (UFTC) | MT535940 |
| 10-001586 | *C. riverinae* | Australia (ANIC) | MT536010 |
| DR 1210.0 | *C. rotundiceps* | Dominican Republic (UFTC) | MT535941 |
| AM11  AM12  KF855854 | *C. secundus* | Australia (ANIC)  Australia (ANIC)  GenBank | MT535924  MT535925  KF855854 |
| 10-001694 | *C. simulatus* | Australia (ANIC) | MT536013 |
| DR 1289.0 | *C. spathifrons* | Dominican Republic (UFTC) | MT535936 |
| PR 207.0 | *C. undulans* | U.S. Territory (UFTC) | MT535943 |
| NA  NA | *Incisotermes minor*  Pellets and adult | USA  FFPRI Japan | HM542456  NA |
